# Image-level trajectory inference of tau pathology using variational autoencoder for Flortaucipir PET

**DOI:** 10.1007/s00259-021-05662-z

**Published:** 2022-02-28

**Authors:** Jimin Hong, Seung Kwan Kang, Ian Alberts, Jiaying Lu, Raphael Sznitman, Jae Sung Lee, Axel Rominger, Hongyoon Choi, Kuangyu Shi, the Alzheimer’s Disease Neuroimaging Initiative

**Affiliations:** 1 Department of Nuclear Medicine, Inselspital, University of Bern, Freiburgstrasse 18, 3010 Bern, Switzerland; 2 Department of Nuclear Medicine, Seoul National University Hospital, 28 Yeon Gun, Jong Ro, Seoul, Republic of Korea; 3 PET Center, Huashan Hospital, Fudan University, Shanghai, China; 4 ARTORG Center, University of Bern, Bern, Switzerland; 5 Department of Informatics, Technical University of Munich, Munich, Germany

**Keywords:** Variational auto-encoder (VAE), Hierarchical agglomerative clustering, Minimum spanning tree (MST), Positron emission tomography (PET), [^18^F]Flortaucipir, Alzheimer’s disease

## Abstract

**Purpose:**

Alzheimer’s disease (AD) studies revealed that abnormal deposition of tau spreads in a specific spatial pattern, namely Braak stage. However, Braak staging is based on post mortem brains, each of which represents the cross section of the tau trajectory in disease progression, and numerous studies were reported that do not conform to that model. This study thus aimed to identify the tau trajectory and quantify the tau progression in a data-driven approach with the continuous latent space learned by variational autoencoder (VAE).

**Methods:**

A total of 1080 [^18^F]Flortaucipir brain positron emission tomography (PET) images were collected from the Alzheimer’s Disease Neuroimaging Initiative (ADNI) database. VAE was built to compress the hidden features from tau images in latent space. Hierarchical agglomerative clustering and minimum spanning tree (MST) were applied to organize the features and calibrate them to the tau progression, thus deriving *pseudo-time*. The image-level tau trajectory was inferred by continuously sampling across the calibrated latent features. We assessed the *pseudo-time* with regard to tau standardized uptake value ratio (SUVr) in AD-vulnerable regions, amyloid deposit, glucose metabolism, cognitive scores, and clinical diagnosis.

**Results:**

We identified four clusters that plausibly capture certain stages of AD and organized the clusters in the latent space. The inferred tau trajectory agreed with the Braak staging. According to the derived *pseudo-time*, tau first deposits in the parahippocampal and amygdala, and then spreads to the fusiform, inferior temporal lobe, and posterior cingulate. Prior to the regional tau deposition, amyloid accumulates first.

**Conclusion:**

The spatiotemporal trajectory of tau progression inferred in this study was consistent with Braak staging. The profile of other biomarkers in disease progression agreed well with previous findings. We addressed that this approach additionally has the potential to quantify tau progression as a continuous variable by taking a whole-brain tau image into account.

**Supplementary Information:**

The online version contains supplementary material available at 10.1007/s00259-021-05662-z.

## Introduction

Neurofibrillary tangles (NFTs) are one of the key pathophysiologic features of Alzheimer’s disease (AD). NFTs are formed by the hyperphosphorylation and abnormal aggregation of tau protein [1]. The abnormal tau pathology is related to cognitive dysfunction, and it predicts longitudinal change in neuronal loss [2, 3]. Therefore, the degree of tau pathology is important to understanding disease progression and may be reflective of clinical severity [4].

Post-mortem data indicate that NFTs follow a distinctive spreading pattern during disease progression when they are distributed, namely Braak stages. In the first two stages (I–II), NFTs appear in the transentorhinal region, then through the limbic region (stages III–IV), and finally in the isocortical region (stages V–VI) [5]. However, Braak staging is based on the autopsy of the half brain which shows a certain cross section of the tau trajectory in disease progression. Recent advances in positron emission tomography (PET) tracers for tau imaging made it possible to assess regional tau load in vivo, which is now a key diagnostic biomarker for diagnosing AD [6]. Of these radiotracers, [^18^F]flortaucipir, also known as [^18^F]AV-1451, has been studied widely and recently approved by FDA [7]. Although numerous [^18^F]flortaucipir PET studies corroborate the Braak staging [8–10], other studies argue otherwise [11–13]. For instance, the tau burden in medial temporal regions has also been addressed in cognitively normal (CN) subjects [14]. Additionally, the clinical variants of AD affect tau deposition patterns [15–20].

Due to the region-specific and variable patterns of tau PET, there is little consensus on how to quantify tau PET scans and incorporate them into the A/T/N scheme—a recommended category of AD biomarkers (A: β-amyloid biomarkers; T: tau biomarkers; N; neurodegeneration or neuronal injury biomarkers) [6]), as the region-of-interest (ROI) can be defined in various ways, and this can significantly influence study outcomes [21, 22]. The most agreed upon method for tau quantification is measuring the standardized uptake value ratio (SUVr) in the predefined ROI, assuming that the topography of tau PET deposition matches well with the Braak stage [8]. The ROIs vulnerable to AD, in this sense, include the entorhinal, amygdala, parahippocampal, fusiform, inferior temporal, and middle temporal regions [23, 24]. Likewise, some studies combined AD-vulnerable ROIs for the quantification and classified tau in multi-stages rather than merely rating it as positive/negative [9, 25, 26]. However, with accumulated evidence of the various spatiotemporal tau patterns and trajectories, a simple region-specific quantification of tau PET can be considered to be less effective [11–20].

Here, we propose a data-driven model to infer an image-level spatial progression pattern of tau pathology in AD. Additionally, we maintain that this method has the potential to quantify the degree of tau deposition as a continuous value by taking the whole-brain tau image into account. We hypothesized [1] that latent, or hidden, patterns inherently exist in each tau PET scan, which represents the cross section of tau progression and [2] that the progression of tau pathology could be modeled by a graph theory. More specifically, we employed the variational autoencoder (VAE), which is an unsupervised generative model [27], to derive the latent features. We used hierarchical agglomerative clustering and minimum spanning tree (MST) to build a trajectory upon these latent features, and to mark the extent of tau progression, which we refer to as *pseudo-time* in this work. With regard to the tau PET-based clusters that were discovered, and the *pseudo-time* that was derived, we analyzed the tau SUVr in AD-vulnerable regions, amyloid deposit, glucose metabolism, cognitive scores, and clinical diagnosis.

## Material and methods

### Data acquisition

Data used in the preparation of this article were obtained from the Alzheimer’s Disease Neuroimaging Initiative (ADNI) database (adni.loni.usc.edu). The ADNI was launched in 2003 as a public–private partnership, led by Principal Investigator Michael W. Weiner, MD. The primary goal of ADNI has been to test whether serial magnetic resonance imaging (MRI), positron emission tomography (PET), other biological markers, and clinical and neuropsychological assessment can be combined to measure the progression of mild cognitive impairment (MCI) and early Alzheimer’s disease (AD). For up-to-date information, see www.adni-info.org. In total, 1080 pairs of T1 MRI images and [^18^F]Flortaucipir PET scans were recruited. Of those pairs, 78 were clinically diagnosed as AD, 483 were diagnosed as MCI, and 519 were diagnosed as CN at the visit closest to tau PET. Inclusion and exclusion criteria can be found at adni.loni.usc.edu. The time window between the PET images and corresponding T1 MRI images was typically within 3 months, but in case that a concurrent MRI was not available, we collected the one from the visit closest to the corresponding tau PET scan. The demographics of the subjects are summarized in Table 1.

**Table 1:** The demographics of the subjects

	AD (*n* = 78)	MCI (*n* = 483)	CN (*n* = 519)	Total (*n* = 1080)
Age (years)	74.8 ± 8.4	74.7 ± 7.4	74.4 ± 7.7	74.6 ± 7.6
Sex (M:F)	46:32	254:229	216:303	516:564
APOE4 * (positive %)	61.9	43.8	36.1	44.6
MMSE**	21.9 ± 4.3	27.5 ± 3.2	28.9 ± 1.4	27.8 ± 3.2
Education ( years)	15.3 ± 2.6	16.4 ± 2.5	16.6 ± 2.4	16.4 ± 2.5

^*^Apolipoprotein E. We defined the subject carrying either APOE3/4 or APOE4/4 as positive case

^**^Mini–Mental State Examination (MMSE)

### Preprocessing

All images underwent preprocessing in the ADNI pipeline. Six 5-min frames of PET were averaged and reoriented into a standard 160 × 160 × 96 voxel image grid, having 1.5-mm^3^ cubic voxels. Each image set was filtered with a scanner-specific filter function to produce images of a uniform isotropic resolution of 8-mm FWHM. Each [^18^F]Flortaucipir PET image was co-registered to the corresponding T1 MRI image by applying a normalized mutual-information-based rigid registration. MRI images were then spatially normalized to a T1 MR template by matching the Montreal Neurological Institute (MNI) space using statistical parametric mapping (SPM8, www.fil.ion.ucl.ac.uk/spm). Combining the transformation matrices of the PET-to-MRI rigid registration and the MRI-to-MNI elastic registration space, the PET images were, finally, spatially normalized into the MNI template. Each PET image was divided by mean tau uptake of the cerebellum and normalized by maximum intensity before being fed into the model.

### Variational autoencoder (VAE)

**Fig. 1:**
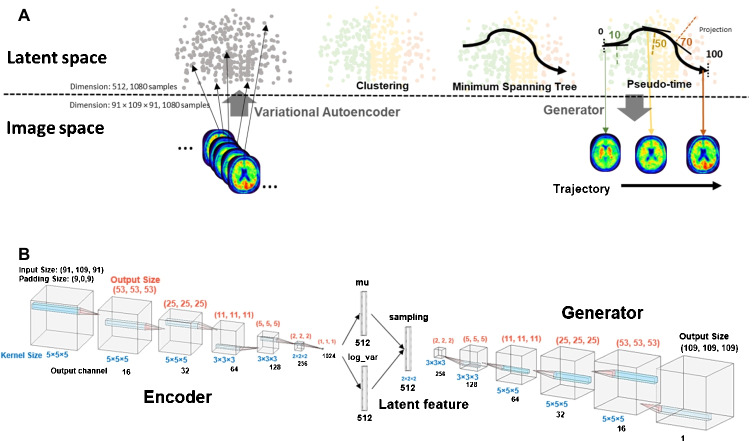
Study design and model architect for variational autoencoder (VAE).** A** The scheme of the study design. The tau brain images were first embedded into the latent feature by VAE, and those latent features were clustered by hierarchical agglomerative clustering. The identified clusters were organized by minimum spanning tree (MST), and the tau trajectory was reproduced with the VAE generator by continuously sampling across the MST graph. *Pseudo-time* was defined to mark the degree of the tau progression. **B** VAE architecture. Six convolving layers were built for both encoder and generator, with the latent size of 512. The numbers in red and blue indicate the output size and kernel size of each layer, respectively. The dimension in direction of width, height, and depth was identical for each kernel and output. The output number of channels was specified in black

The conceptual design and method used for each step are illustrated in Fig. 1A and explained in the Supplementary Material. VAE was exploited to encode the [^18^F]Flortaucipir PET images into the latent space and to generate the tau progression images inferred from the latent space. VAE is a technique that reduces dimensionality; it compresses the input data into concentrated features, or latent features, in a smaller dimension. However, contrary to independent component analysis (ICA), principal component analysis (PCA), or standard autoencoder (AE), VAE encodes the input data as a distribution instead of a point and regularizes the latent space by limiting the distribution produced by the encoder to be Gaussian. Therefore, VAE can generate a new instance by sampling from the continuous latent space, which is composed of a mixture of distributions.

In this work, the encoder and generator were built using six convolutional layers, and the latent feature dimension is of size 512, as illustrated in Fig. 1B. As an unsupervised model, VAE was trained by half of the randomly sampled dataset (540 total subjects; 30 AD, 264 CN, and 246 MCI) for 100 epochs, and it was applied to the entire dataset. Meanwhile, the other half of the dataset was set aside to assess overfitting in the model.

### Latent feature clustering analysis

The latent features of each 1080 [^18^F]Flortaucipir PET scan were categorized by using hierarchical agglomerative clustering. The clustering result was plotted by T-distributed stochastic neighbor embedding (t-SNE), which enables the latent feature in a dimension of 512 to be visualized in 2 dimensions.

The AD-vulnerable region tau SUVr, with cerebellum gray matter as a reference region, was evaluated between the clusters. The regions included the inferior temporal lobe (temporal_inf), amygdala, parahippocampal, hippocampus, fusiform, and posterior cingulum (cingulum_post), as defined by Automated Anatomical Labeling (AAL) atlas.

In addition, the age, Mini-Mental Status Examination (MMSE) score, [^18^F]AV45 SUVr, [^18^F]FDG SUVr, and APOE4 status were analyzed between the clusters, all of which were collected from the ADNI data archive for each subject. The MMSE score, the [^18^F]AV45, and the [^18^F]FDG PET SUVr were investigated to compare global cognitive function, amyloid load, and brain metabolism, respectively. [^18^F]AV45 SUVr is defined as the mean SUVr of ROIs, including frontal, anterior cingulate, precuneus, and parietal cortex, with the whole cerebellum outlined by Freesurfer as a reference region [28]. Similarly, [^18^F]FDG PET quantification data were calculated by the average [^18^F]FDG SUVr of angular, temporal, and posterior cingulate, normalized by pons/vermis reference region mean [29].

The significance cluster difference was evaluated by using chi-squared analysis for the categorical clinical phenotype variables. One-way analysis of variance (ANOVA) was performed for continuous clinical phenotype variables, followed by Tukey’s post hoc pairwise test for multiple comparisons.

### Trajectory inference using minimum spanning tree (MST)

The clusters we identified were thought to represent a certain stage in tau progression, and MST was performed to find the connection between or the order of clusters. MST is defined as a weighted, undirected subset of edges in a network that connects all nodes with the minimum possible total edge weight without cycles.

In this work, we assumed that the clusters adjacent to one another in latent space shared more features of tau pathology than the ones which were not adjacent. We also assumed that the progression from one cluster to another was more likely to take place in the direction where the total distance was the shortest. Accordingly, we applied MST in latent space, by defining nodes as the centers of the identified clusters, and edge weights as the Euclidean distance between the corresponding pair of cluster centers.

The MST graph we derived was used as a calibration to measure the tau progression, corresponding the nodes to the marks and the edge weight to the distances between the marks. The latent features were sampled continuously across the edges of the resulting MST graph in order of tau progression, and the image-level tau trajectory was inferred by the trained VAE generator. The resulting tau trajectory was evaluated with regard to the Braak stages that were drawn from Desikan Killiany atlas, which is specified in Supplementary Material.

### Pseudo-time

In addition, we defined the *pseudo-time* of each tau image. The *pseudo-time* describes the extent of tau progression as a continuous value in a range of 0–100, which is measured by the geometrical relationship between its latent feature and the MST graph derived in latent space. For each tau image, we first calculated its latent vector relative to its cluster center. Then, we projected this vector onto the corresponding edge in the MST graph and measured the scalar projection to pinpoint the location. The points marked by each latent feature were normalized and scaled in a range of 0–100, which we refer to as *pseudo-time.*

The regional tau uptake and various biomarker information, retrieved from the ADNI data archive, were outlined with regard to the *pseudo-time* that was derived. In order to estimate the *pseudo-time* at which SUVr of regional tau PET, as well as other biomarker PET, significantly changes, we regressed the *pseudo-time* SUVr curves to the logistic model using SPSS Statistics for Windows, version 23. 0 (SPSS Inc., Chicago, IL, USA), which is described in detail in Supplementary Fig. 1.

Moreover, we calculated the length between each latent feature and the respective edge in the MST graph, and we flagged the outliers that fell outside the 95 percentile in order to potentially detect the atypical tau patterns. The tau patterns of the outliers were examined.

## Results

### Cluster identifications

**Fig. 2:**
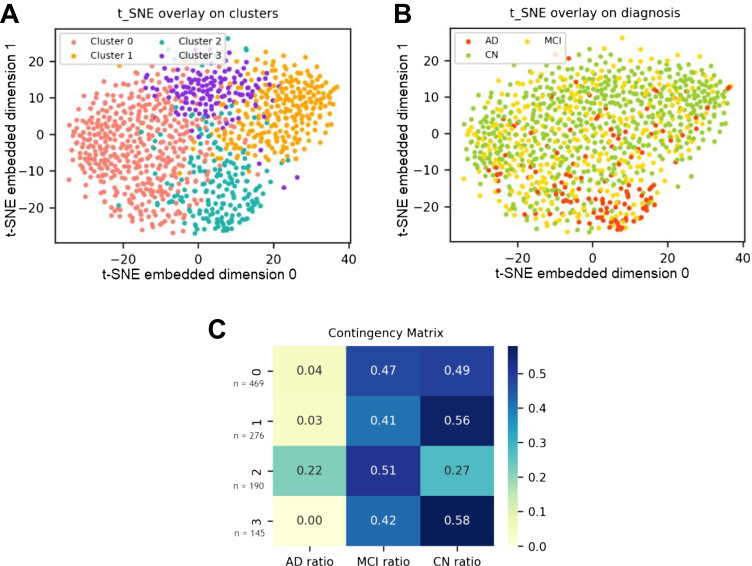
Clustering results on t-SNE plot and contigency table.**A** t-SNE plot with clustering result. **B** t-SNE plot with diagnosis. **C** Contingency matrix of cluster result and the diagnosis ratio

We identified four clusters from the latent features encoded by VAE and hierarchical agglomerative clustering. The t-SNE plot in Fig. 2A–B illustrates the clustering result and the diagnosis of 1080 data, respectively. The heatmap of the contingency table is shown in Fig. 2C, which depicts the diagnosis ratio within each cluster. AD subjects were not found in cluster 3, whereas they were prevalent in cluster 2. MCI subjects were found mostly in clusters 0 and 2. There was a subtle difference in ratios between clusters 1 and 3.

### Standardized uptake value ratio (SUVr) in ROI between clusters and Braak stages

**Fig. 3:**
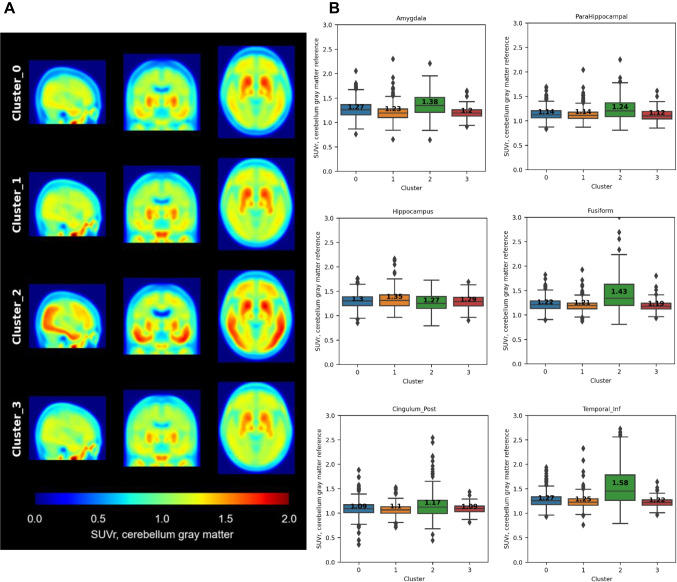
Average tau PET image of each cluster. **A** The average image of each cluster. **B** Tau SUVr in temporal and cingulate region (amygdala, parahippocampal, hippocampus, fusiform, cingulum_post, and temporal_inf)

Figure 3A depicts the average tau PET image of each cluster, normalized by average uptake in cerebellum gray matter. On average, tau deposition gradually increased in temporal lobe and frontal lobe in this order: cluster 3, cluster 1, cluster 0, and cluster 2. Figure 3B illustrates the tau SUVr within AD-vulnerable ROIs. Across AD-vulnerable regions, cluster 2 presented the highest average SUVr, except for hippocampus, where cluster 1 showed the highest value. Supplementary Table 1 shows the results from ANOVA and Tukey’s pairwise test in temporal and cingulate ROIs as defined in AAL template. In most ROIs, the group difference between cluster 0, cluster 1, and cluster 3 was trivial, except for amygdala, where cluster 0 presented significantly more tau deposition than cluster 3. Thus, cluster 2 was regarded as a typical AD with advanced tau depositions, while the other three clusters represented normal or early tau pathology progression.

### Clinical variance between the clusters

Table 2 summarizes the clinical variance between the clusters. The average age was lowest in cluster 3, and no pronounced difference in the average age was found between other clusters. The average MMSE score was lowest in cluster 2, and second-lowest in cluster 0, but no difference was found between clusters 1 and 3. The average [^18^F]AV45 PET SUVr was highest in cluster 2, but no significant difference was found between the other three clusters. The average [^18^F]FDG PET SUVr was lowered in the following order: cluster 3, cluster 1, cluster 0, and cluster 2. However, Tukey’s test indicated no difference between clusters 1 and 3. APOE4-positive cases were prevalent in cluster 2, and less common in cluster 3, followed by clusters 0 and 1. Female subjects were more included in cluster 3 and less included in cluster 0.

**Table 2:** The statistical differences of age, MMSE, [^18^F]AV45, FDG, sex, and APOE status between each cluster

	Age	MMSE	[^18^F]AV45	[18F]FDG	Sex (F)	APOE (positive)
Cluster_0	74.79 ± 7.16	28.06 ± 2.72	1.14 ± 0.20	1.22 ± 0.14	43.92%	38.76%
Cluster_1	75.03 ± 7.84	28.56 ± 2.02	1.13 ± 0.19	1.27 ± 0.11	59.42%	36.07%
Cluster_2	76.13 ± 8.11	24.98 ± 4.66	1.37 ± 0.25	1.09 ± 0.12	51.57%	52.94%
Cluster_3	70.77 ± 6.64	28.95 ± 1.19	1.11 ± 0.17	1.30 ± 0.10	66.20%	43.79%
Test value (*F*/chi^2^)	15.78	74.12	31.96	26.04	30.07	13.88
*p* value	< 0.001	< 0.001	< 0.001	< 0.001	< 0.001	< 0.01
Tukey’s test significance	[0,3], [1–3]	[0,2], [0,3], [1–3]	[0,2], [1–3]	[0,2], [0,3], [1–3]		

### MST and inference of tau trajectory

**Fig. 4:**
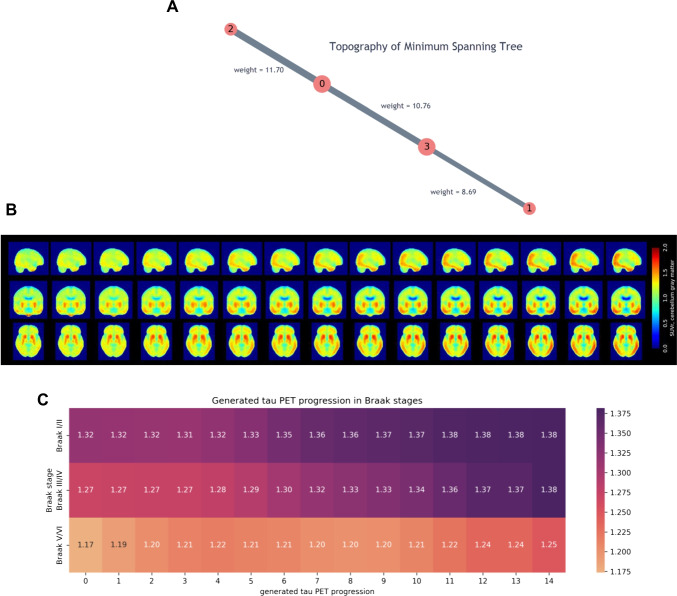
Generation of a movie for tau progression pattern using VAE and MST.**A** MST graph. The red point depicts the center of each cluster. The gray line illustrates the edge with the edge weight. **B** Tau progression pattern (left to right) generated by the derived MST graph and the trained VAE generator in sagittal (top), coronal, and axial (bottom) view. **C** The heatmap of generated tau PET progression in Braak stages

The MST method resulted in a straight graph, with clusters 1 and 2 as leaf nodes, as shown in Fig. 4A. Although MST is an undirected graph and does not provide the starting and endpoint of the network, we determined cluster 1 as the initial node of tau progression, and cluster 2 as the last node, given the regional tau uptake and the clinical variance between the clusters. Accordingly, the path of cluster 1, cluster 3, cluster 0, and cluster 2 was established as a tau progression sequence. The latent vector trajectory was derived by continuously sampling the edges through the defined MST graph, and the tau PET trajectory was reproduced by using the VAE generator, as shown in Fig. 4B. The video is provided in Supplementary Materials. Similar to Braak stages, the tau deposits initially in the medial temporal lobe and gradually in the temporal and frontal lobes, as illustrated in Fig. 4C. The average SUVr gradually increased in ROIs related to Braak stages I/II, followed by ROIs associated with Braak stages III/IV and V/VI. The average SUVr changed at a rate that was greater in ROIs related to Braak stages III/IV than in Braak stages I/II and V/VI. The smallest increase of average SUVr was found in ROIs related to Braak stages V/VI.

### Pseudo-time analysis

**Fig. 5:**
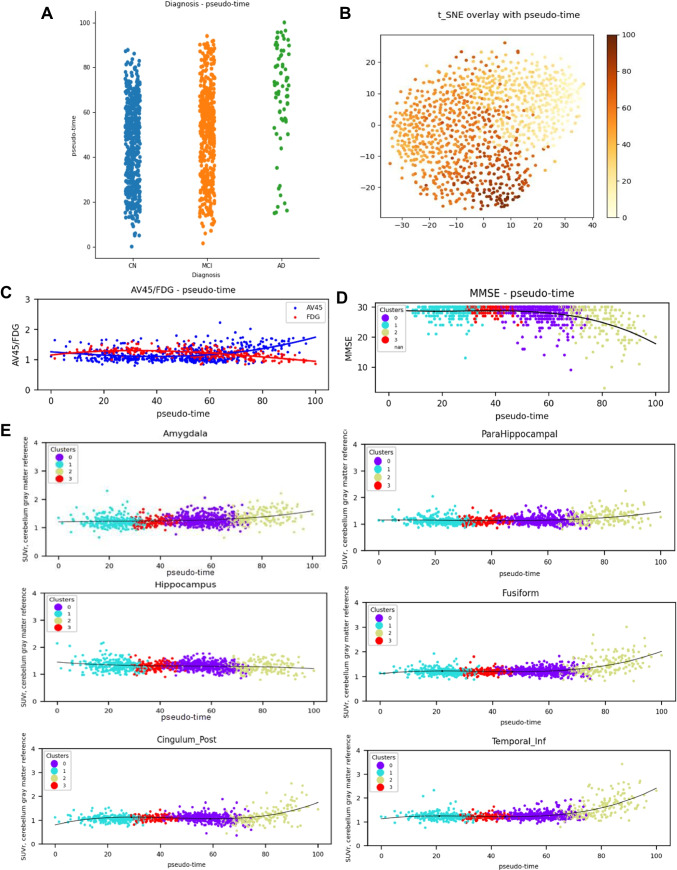
Pseudo-time vs diagnosis/SUVr. **A** *Pseudo-time* vs diagnosis. **B** t-SNE plot of *pseudo-time*. **C** *Pseudo-time* vs [^18^F]AV45 /FDG. **D.** *Pseudo-time* vs MMSE. **E** *Pseudo-time* vs tau SUVr in amygdala, hippocampus, parahippocampal, fusiform, temporal_inf, cingulum_post. The scatter plot was fitted to third-degree polynomial (black line)

The *pseudo-time* was measured for each tau image. Figure 5A depicts the *pseudo-time* between different diagnosis groups. The *pseudo-time* in the AD group was greater than in the CN group, while the *pseudo-time* in the MCI group showed a broader range. Figure 5B illustrates the derived *pseudo-time* in the t-SNE plot. Figure 5C depicts the *pseudo-time* profile for amyloid deposit estimated by [^18^F]AV45 PET and for glucose metabolism estimated by [^18^F]FDG PET. The amyloid deposit gradually increased, whereas glucose metabolism stayed stable. Figure 5D shows the MMSE score, and Fig. 5E illustrates tau SUVr profiles in AD-vulnerable ROIs in terms of *pseudo-time*. The profile of fusiform, cingulum_post, and temporal_inf appeared similar, where a drastic upturn was found in the *pseudo-time* tail. The outline of hippocampus stayed unchanged, but that of amygdala and parahippocampal consistently increased. The MMSE declined in around a *pseudo-time* of 60. The logistic regression of Fig. 5C,E is illustrated in Supplementary Fig. 1. The amyloid deposit calculated by [^18^F]AV45 PET increased at first, and the tau SUVr in AD-vulnerable ROIs later escalated. More specifically, the tau SUVr started to increase first in the parahippocampal, and then in amygdala, fusiform, temp_inf, and cingulum_post. However, the tau SUVr of hippocampus stayed rather consistent, and [^18^F]FDG SUVr increased.

In addition, the tau patterns of the outliers were acquired, which showed visually atypical tau patterns; the examples are introduced in Supplementary Fig. 2. In total, 4.9% of cluster 0, 3.2% of cluster 1, and 11.5% of cluster 2 were flagged as atypical*.* With our method, we found no outliers in cluster 3.

## Discussion

For decades, the trajectory of tau pathology has been primarily studied upon a single model—Braak stages—despite numerous cases being reported that do not conform to that model. Besides the fact that the model is region specific, these variants of tau trajectories added extra challenges to quantify the progress of tau in AD. Recently, the subtypes and trajectories of tau deposition were extensively studied [20]. In parallel, efforts were made to find the best quantification scheme to account for tau PET scans in AD [23, 24]. In this study, we employed a deep learning algorithm to infer the tau trajectory without any prior knowledge. We thus found the *pseudo-time* of tau progress, which quantifies the extent of tau deposit in disease progression at the individual level. Our finding reassured Braak’s model, while demonstrating that *pseudo-time* potentially solves the challenges in tau quantification previously observed. Our approach takes the whole brain tau PET itself into consideration and delivers the continuous tau progress index.

When building the tau trajectory, we detected outliers that showed visually *atypical* tau patterns. The *atypical* pattern mainly included a severe asymmetricity, as illustrated in Supplementary Fig. 2. The outliers were found most frequently in cluster 2, which represents the later stage of tau progression as tau progresses through cluster 1, cluster 3, cluster 0, and cluster 2. Aside from the *typical* presentation of tau pathology from the medial temporal regions and the resulting impairment of episodic memory, studies have found *atypical* tau patterns and their related clinical symptoms, such as primary progressive aphasia (PPA) or posterior cortical atrophy (PCA) [30, 31]. On the other hand, a recent study identified the four distinctive trajectories of tau pathology with rather similar prevalence and suggested re-examining the notion of “typical AD” [20]. Yet, in this work, we focused on deriving the *typical* tau trajectory and flagged the resulting outliers as simply *atypical* tau patterns. However, we believe that our method can contribute to the extensive research on *atypical*, if not, various, tau trajectories and their related symptoms.

The priori ROI-based quantification faces challenges. One of them is its limitation in reflecting the various and atypical spatial patterns of tau accumulation in PET [30, 31]. Because the progression of tau is non-linear, ROI-based quantification in tau PET is less efficient to capture the subtle changes within the predefined ROIs. Furthermore, image noise and quantification errors hinder the performance of the ROI-based quantification method at the early stage of tau pathology with mild tau accumulation. Our method dealt with this concern by employing the renowned deep learning algorithm, VAE, and by using a whole-brain tau image as an input. In our study, VAE served as the model to learn non-linear dimension reduction functions of higher dimension PET images. The latent vectors in autoencoder (AE) tend to be uninterpretable as AE finds the latent vector which merely reconstructs the input as similarly as possible. In contrast, VAE encodes the latent features by regularizing the latent space to be continuous, hence making it possible to generate new data by sampling from its latent space. The progression of tau pathology was then modeled upon continuous latent features by applying hierarchical agglomerative clustering and MST. This occurred such that adjacent latent features share a similar tau pathology, and the progression takes place where the sum of distances between latent clusters is minimal. One possible direction of future work is to modify the modeling of tau progression with help from the longitudinal follow-up images from subjects.

Moreover, deriving the *pseudo-time* can help to better understand the complementary information of tau regional uptake and various PET biomarkers, including amyloid pathology and brain metabolism. For instance, our finding supports the fact that the accumulation rate, especially in inferior temporal lobe, can be a good alternative indicator, as was also suggested by a longitudinal tau PET study from another group [12]. Before the tau uptakes take place regionally, amyloid PET uptake starts increasing first. This consequence of biomarker corresponds to the previously well-known hypothesis of dynamic biomarkers of AD pathological cascade [32, 33]. However, the *pseudo-time* for hippocampus is poorly estimated. We speculate that the misleading result might come from brain atrophy. Similarly, the logistic regression for the FDG *pseudo-time* curve is unclear, as it increases rather than decreases throughout *pseudo-time.* We believe that one possible cause for this is the lack of information in ADNI. That is, the distribution of the FDG p*seudo-time* curve was comparatively sparse for logistic regression. In addition, the inclusion of the amyloid-negative subjects in this study might interfere with the exploration of FDG cascade. The conflicting relationship between tau and glucose metabolism has been reported in amyloid negative MCI, where FDG hypermetabolism was associated with higher tau PET [34]. However, apart from results indicated by logistic regression, [^18^F]FDG SUVr decreases in late *pseudo-time*, as illustrated in Fig. 5C and was the lowest in cluster 2 which is the last cluster in *pseudo-time* as seen in Table 2. Thus, we believe that FDG was associated with *pseudo-time*, but the relation may occur outside the *pseudo-time* range, which is consistent with the conventional sequence of biomarkers: amyloid and tau, followed by decreasing global metabolism.

A limitation in our study also comes from the data itself. In particular, the first-generation [^18^F]Flortaucipir has limited sensitivity to tau in the early stage [35, 36], which restricted the characterization of our method, especially in the early stages. This issue might have contributed to the relative lack of statistically significant differences observed for the regional tau SUVr between the early clusters (0, 1, 3) in some of the ROIs we considered, as shown in Supplementary Table 1. Also, the data were not corrected from the partial volume effect, which restricts the analysis related to SUVr in smaller regions, such as amygdala, hippocampus, and parahippocampal in the earlier stage [37]. For instance, the lower pseudo R squared values were observed in amygdala, hippocampus, and parahippocampal, in comparison to other ROIs as shown in Supplementary Fig. 1. Furthermore, as measure of specific binding, the SUVr that was used to assess the *pseudo-time* may be biased as it is dependent on the changes in flow, tracer uptake, and clearance rates [38, 39].

The methodological limitation remains in each building block of this study. Although the VAE worked well with our purposes, the outputs generated from the VAE for trajectory analysis were blurry, which is known to be common [40]. Another limitation for this work is the heuristic setting in the number of resulting clusters. That is, we fixed the number of clusters manually in order to avoid overfitting and underfitting problems, referring to the dendrogram, distance plot, t-SNE plot, and diagnosis contingency matrix. MST has been a popular choice in brain network or connectivity studies, mostly in fMRI studies [41, 42], and it was recently introduced in AD disease progression study by using gene expression data [43]. To the best of our knowledge, MST has not yet been explored in brain PET imaging to model the trajectory of disease progression in AD. In our study, MST showed the potential to discover the relationship between the tau PET clusters, hence, the tau trajectory. Furthermore, we observed the nested sub-network in MST with a larger number of clusters. This can indicate that if our method is expanded to a larger number of clusters, the distinctive subtypes of the tau trajectories can be identified together with their *pseudo-time*. However, as an undirected graph, the start and end points are not determined by MST. This opens up more place for discussion about the interpretation of the result derived by a larger number of clusters.

## Conclusion

In this work, we identified the image-level tau trajectory without any prior knowledge of existing models such as Braak stages. We did so by using VAE, hierarchical agglomerative clustering, and MST. Additionally, we suggested that this approach has the potential to quantify tau progress as a continuous value, which we refer to as *pseudo-time*. In contrast to the ROI-based quantification method, our approach considers the whole brain image to pinpoint the extent of tau progress at the individual level. Furthermore, *pseudo-time* can guide the better understanding of the association between tau burden and other biomarkers such as amyloid, neuronal injury in the pathophysiology of AD. Importantly, atypical tau patterns were detected upon deriving tau trajectory, and we believe this approach sheds new light on an extensive search for distinctive tau trajectories as well as their quantification.

## Supplementary Information

Below is the link to the electronic supplementary material.

Supplementary file1 (DOCX 3.52 MB)

Online Resource 2 (MP4 12.2 MB)

## Data Availability

Data collection and data sharing for this project were funded by the Alzheimer’s Disease Neuroimaging Initiative (ADNI) (National Institutes of Health Grant U01 AG024904) and by DOD ADNI (Department of Defense award number W81XWH-12–2-0012). ADNI is funded by the National Institute on Aging, the National Institute of Biomedical Imaging and Bioengineering, and through generous contributions from the following: AbbVie, Alzheimer’s Association; Alzheimer’s Drug Discovery Foundation; Araclon Biotech; BioClinica, Inc.; Biogen; Bristol-Myers Squibb Company; CereSpir, Inc.; Cogstate; Eisai Inc.; Elan Pharmaceuticals, Inc.; Eli Lilly and Company; EuroImmun; F. Hoffmann-La Roche Ltd and its affiliated company Genentech, Inc.; Fujirebio; GE Healthcare; IXICO Ltd.; Janssen Alzheimer Immunotherapy Research & Development, LLC.; Johnson & Johnson Pharmaceutical Research and Development LLC.; Lumosity; Lundbeck; Merck & Co., Inc.; Meso Scale Diagnostics, LLC.; NeuroRx Research; Neurotrack Technologies; Novartis Pharmaceuticals Corporation; Pfizer Inc.; Piramal Imaging; Servier; Takeda Pharmaceutical Company; and Transition Therapeutics. The Canadian Institutes of Health Research is providing funds to support ADNI clinical sites in Canada. Private sector contributions are facilitated by the Foundation for the National Institutes of Health (www.fnih.org). The grantee organization is the Northern California Institute for Research and Education, and the study is coordinated by the Alzheimer’s Therapeutic Research Institute at the University of Southern California. ADNI data are disseminated by the Laboratory for Neuro Imaging at the University of Southern California.
