# Supplementary material for: Image-level trajectory inference of tau pathology using variational autoencoder for Flortaucipir PET
Source: Eur J Nucl Med Mol Imaging. 2022 Feb 28;49(9):3061–72. doi: 10.1007/s00259-021-05662-z (PMC9250490; doi:10.1007/s00259-021-05662-z)
Supplement: Supplementary file 1 — Supplementary file1 (DOCX 3.52 MB) [file 259_2021_5662_MOESM1_ESM.docx]

**Supplementary Material**

**Variational autoencoder (VAE)**

Given the data sample projected in the latent space, z, the probabilistic generator can be expressed as $p_{Ɵ}\left( x \right| z)$. The posterior distribution $p_{Ɵ}\left( z \right| x)$ is obtained by using the prior distribution $p(z)$ and the probabilistic generator $p_{Ɵ}\left( x \right| z)$, such that $p_{Ɵ}\left( z \right| x) \sim p(z)p_{Ɵ}\left( x \right| z).$ The encoder learns an approximation $q_{\emptyset}\left( z \right| x)$ to the posterior distribution $p_{Ɵ}\left( z \right| x)$, where$\emptyset$ denotes the parameters of the encoder, and $Ɵ$ those of the generator. Hence, the VAE loss function used to train the model can be described as,

$$L \left( \emptyset, Ɵ \right)=- E_{z \sim q_{\emptyset}\left( z \right| x)}\left( log p_{Ɵ}\left( x \right| z \right) )+KL( q_{\emptyset}\left( z \right| x) |\left| p_{Ɵ}\left( z \right) \right).$$

Note that the first term defines the reconstruction loss, while the second term acts as a regularization term, in the form of the Kullback-Leibler (KL) divergence between the latent distribution learnt and the prior distribution. In practice, the generator input is resampled by the encoded latent features z,

$$z_{resampled}= z_{encoder}+ z_{sd} \times\varepsilon,$$

where $\varepsilon$ represents a random noise sample.

For this work, VAE is trained to embed the latent feature, $z_{tauPET}$, for each brain tau PET, thus creating the distribution of $z_{tauPET}$ in latent space.

**Hierarchical agglomerative clustering**

For the initial step, each $z_{tauPET}$ starts in its own cluster, and the initial cluster distances are calculated as the squared Euclidean distance between $z_{resampled}$ of all possible pairs. The pair with the shortest squared Euclidean distance is merged, thereby updating sets of clusters and cluster centres.

At each step, the pairs of clusters are merged. That minimizes the total within-cluster variance (Ward’s minimum variance criterion), until the desired number of output clusters are met. Therefore, the merging cost of combing the cluster A and B is calculated as follows,

$$\Delta\left( A,B \right)= \sum_{i\in A,B} \left| \left| \vec{x}_{i}- \vec{m}_{A\cup B} \right| \right|^{2}- \sum_{i\in A} \left| \left| \vec{x}_{i}- \vec{m}_{A} \right| \right|^{2}- \sum_{i\in B} \left| \left| \vec{x}_{i}- \vec{m}_{B} \right| \right|^{2}$$

$$= \frac{n_{A}n_{B}}{n_{A}+ n_{B}}\left| \left| \vec{m}_{A}- \vec{m}_{B} \right| \right|^{2}$$

where, $\vec{m}_{i}$ is the centre of cluster i, and $n_{i}$ is the number of objects in cluster i.

**Minimum Spanning Tree (MST)**

Kruskal’s algorithm was used to find the minimum spanning tree. The resulting graph is a connected graph where a subset of edges forms a tree including every vertex, while the sum of the edge weight is kept to the minimum.

| Pseudocode |
| --- |
| Define vertice $\boldsymbol{V}$, and edge $\boldsymbol{E}$.  $\boldsymbol{v}_{\boldsymbol{i}}$ = Cluster center (i = 1,2,…,n)  $\boldsymbol{e}_{\boldsymbol{ij}}$ = Euclidean distance between $\boldsymbol{v}_{\boldsymbol{i}}$ and $\boldsymbol{v}_{\boldsymbol{j}}$  F := ø  For $\boldsymbol{v}_{\boldsymbol{i}} \boldsymbol{\in}\boldsymbol{V}$:  Make-disjoint-set($\boldsymbol{v}$)  Sort $\boldsymbol{E}$ in ascending order  For $\boldsymbol{e}_{\boldsymbol{ij}}\boldsymbol{\in}\boldsymbol{E}$:  If Find-set($\boldsymbol{v}_{\boldsymbol{i}}$) ≠ Find-set($\boldsymbol{v}_{\boldsymbol{j}}$):  F = F U {($\boldsymbol{v}_{\boldsymbol{i}}\boldsymbol{,}\boldsymbol{v}_{\boldsymbol{j}}$):)}  Union($\boldsymbol{v}_{\boldsymbol{i}}\boldsymbol{,}\boldsymbol{v}_{\boldsymbol{j}}$)  Return F |

**Region of interests(ROIs) in Braak stages**

Desikan Killiany cortical atlas was used to derive ROIs related to Braak stages (39).

| **Braak stage I/II** | | **Braak stage III/IV** | | **Braak stage V/VI** | |
| --- | --- | --- | --- | --- | --- |
| Index(Left, Right) | ROI | Index(Left, Right) | ROI | Index(Left, Right) | ROI |
| 1006,2006 | Entorhinal | 1016,2016 | Parahippocampal | 1028,2028 | Superior_frontal |
| 17,53 | Hippocampus | 1007,2007 | Fusiform | 1012,2012 | Lateral_orbitofrontal |
|  |  | 1013,2013 | Lingual | 1014,2014 | Medial_ orbitofrontal |
|  |  | 18,54 | Amygdala | 1032,2032 | Frontal_pole |
|  |  | 1015,2015 | Middletemporal | 1003,2003 | Caudal_middle_frontal |
|  |  | 1002,2002 | Caudantcing | 1027,2027 | Rostral_middle_frontal |
|  |  | 1026,2026 | Rostantcing | 1018,2018 | Pars_opercularis |
|  |  | 1023,2023 | Postcing | 1019,2019 | Pars_orbitalis |
|  |  | 1010,2010 | Isthmuscing | 1020,2020 | Pars_triangularis |
|  |  | 1035,2035 | Insula | 1011,2011 | Lateraloccipital |
|  |  | 1009,2009 | Inferiortemporal | 1031,2031 | Parietalsupramarginal |
|  |  | 1033,2033 | temppole | 1008,2008 | Parietalinferior |
|  |  |  |  | 1030,2030 | Superiortemporal |
|  |  |  |  | 1029,2029 | Parietalsuperior |
|  |  |  |  | 1025,2025 | Precuneus |
|  |  |  |  | 1001,2001 | BankSuperiorTemporalSulcus |
|  |  |  |  | 1034,2034 | Tranvtemp |
|  |  |  |  | 1021,2021 | Pericalcarine |
|  |  |  |  | 1022,2022 | Postcentral |
|  |  |  |  | 1005,2005 | Cuneus |
|  |  |  |  | 1024,2024 | Precentral |
|  |  |  |  | 1017,2017 | Paracentral |

**Supplementary Table 1 The statistical differences of SUVr in temporal and cingulate ROIs between each cluster, in descending order of F value.**

*****The paired clusters in the square brackets shows significance pair-wise group difference.

N.S.: Not significant

|  | Cluster 0 | | Cluster 1 | | Cluster 2 | | Cluster 3 | | F value | p value | *Tukey’s test significance |
| --- | --- | --- | --- | --- | --- | --- | --- | --- | --- | --- | --- |
| **Temporal_Inf** | | 1.27 ± 0.14 | | 1.25 ± 0.22 | | 1.58 ± 0.42 | | 1.22 ± 0.1 | 101.70 | < 0.001 | [0,2],[1,2],[2,3] |
| **Fusiform** | | 1.22 ± 0.13 | | 1.21 ± 0.25 | | 1.43 ± 0.35 | | 1.19 ± 0.11 | 55.07 | < 0.001 | [0,2],[1,2],[2,3] |
| **Temporal_Mid** | | 1.17 ± 0.13 | | 1.18 ± 0.39 | | 1.44 ± 0.41 | | 1.17 ± 0.09 | 49.75 | < 0.001 | [0,2],[1,2],[2,3] |
| **ParaHippocampal** | | 1.14 ± 0.13 | | 1.14 ± 0.27 | | 1.24 ± 0.23 | | 1.12 ± 0.11 | 15.95 | < 0.001 | [0,2],[1,2],[2,3] |
| **Amygdala** | | 1.27 ± 0.17 | | 1.23 ± 0.4 | | 1.38 ± 0.24 | | 1.2 ± 0.13 | 15.93 | < 0.001 | [0,2],[0,3],[1,2],[2,3] |
| **Hippocampus** | | 1.3 ± 0.14 | | 1.35 ± 0.39 | | 1.27 ± 0.19 | | 1.29 ± 0.14 | 4.18 | < 0.01 | [1,2] |
| **Temporal_Sup** | | 1.05 ± 0.1 | | 1.09 ± 0.66 | | 1.14 ± 0.25 | | 1.07 ± 0.08 | 2.69 | < 0.1 | [0,2] |
| **Heschl** | | 1.04 ± 0.1 | | 1.04 ± 0.72 | | 1.02 ± 0.19 | | 1.02 ± 0.08 | N.S | N.S | N.S. |

|  | Cluster 0 | | Cluster 1 | | Cluster 2 | | Cluster 3 | | F value | p value | *Tukey’s test significance |
| --- | --- | --- | --- | --- | --- | --- | --- | --- | --- | --- | --- |
| **Cingulum_Mid** | | 1.1 ± 0.12 | | 1.09 ± 0.38 | | 1.2 ± 0.26 | | 1.1 ± 0.09 | 10.19 | < 0.001 | [0,2],[1,2],[2,3] |
| **Cingulum_Post** | | 1.09 ± 0.15 | | 1.1 ± 0.51 | | 1.17 ± 0.32 | | 1.09 ± 0.1 | 3.51 | < 0.03 | [0,2],[1,2] |
| **Insula** | | 1.14 ± 0.11 | | 1.14 ± 0.63 | | 1.17 ± 0.23 | | 1.11 ± 0.09 | N.S | N.S | N.S. |
| **Cingulum_Ant** | | 1.08 ± 0.12 | | 1.08 ± 0.47 | | 1.07 ± 0.22 | | 1.08 ± 0.1 | N.S | N.S | N.S. |


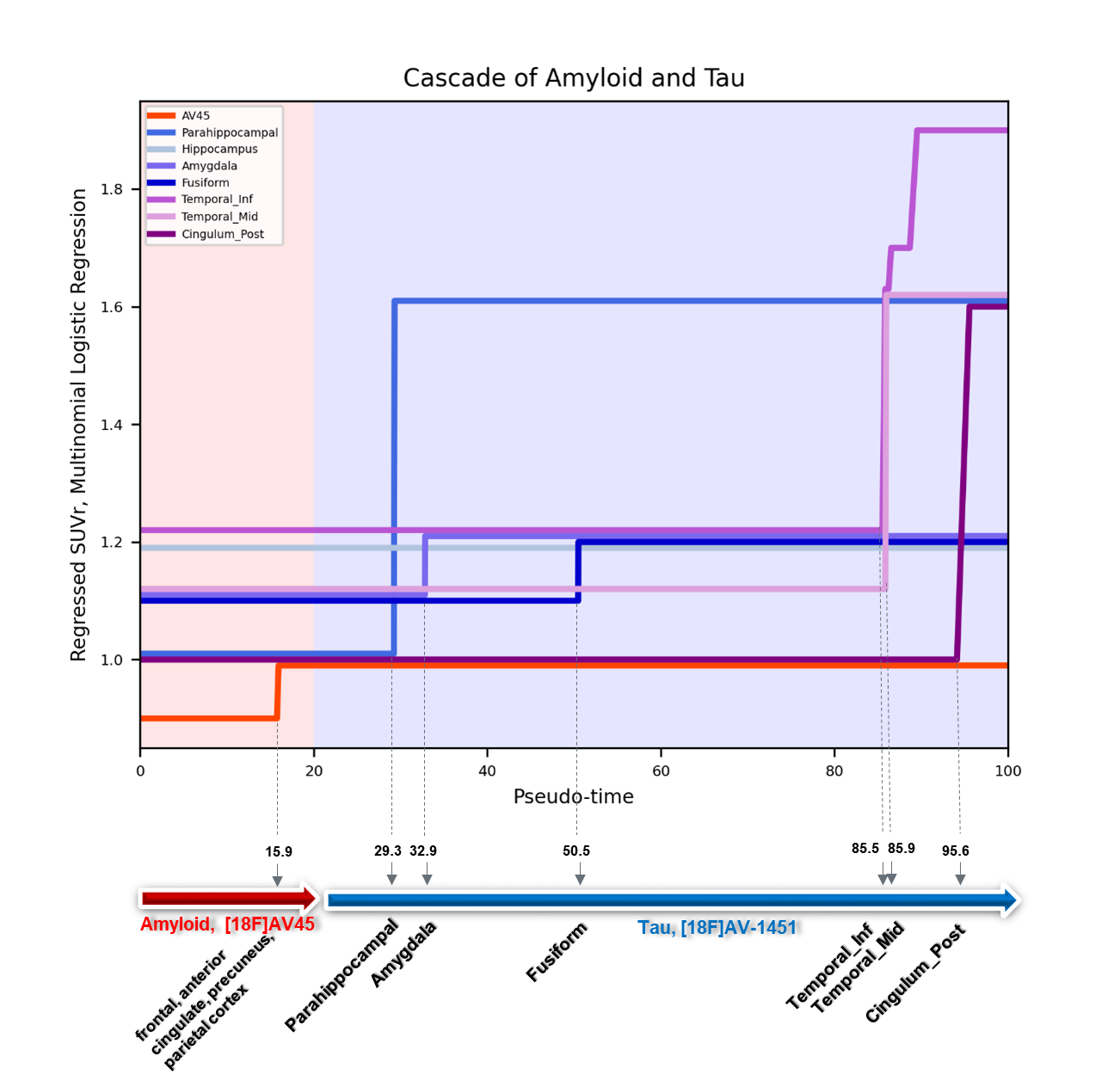


Nagelkerke’s pseudo-R squared

| **Amygdala** | **Parahippocampal** | **Hippocampus** | **Fusiform** | **Temporal_Inf** | **Temporal_Mid** | **Cingulate_Post** |
| --- | --- | --- | --- | --- | --- | --- |
| 0.112 | 0.097 | 0.050 | 0.198 | 0.270 | 0.244 | 0.141 |

**Supplementary Figure 1. Cascade of amyloid and tau in *pseudo-time*.** Multinomial logistic regression was performed to pinpoint the knee of the curve where the SUVr rises significantly. The SUVrs in each ROI were categorized by rounding to one decimal place. SPSS Statistics for Windows, version 23. 0 (SPSS Inc., Chicago, Ill., USA) was used to regress the categorized SUVr to logistic function. [^18^F]AV45 SUVr is defined as the mean SUVr of ROIs, including frontal, anterior cingulate, precuneus, and parietal cortex, with the whole cerebellum outlined by Freesurfer as a reference region (28). [^18^F]AV-1451 SUVr of hippocampus stayed consistent within the *pseudo-time* we derived. The table shows Nagelkerke’s R^2^ to account for the proportion of the total variability explained by the regression model.


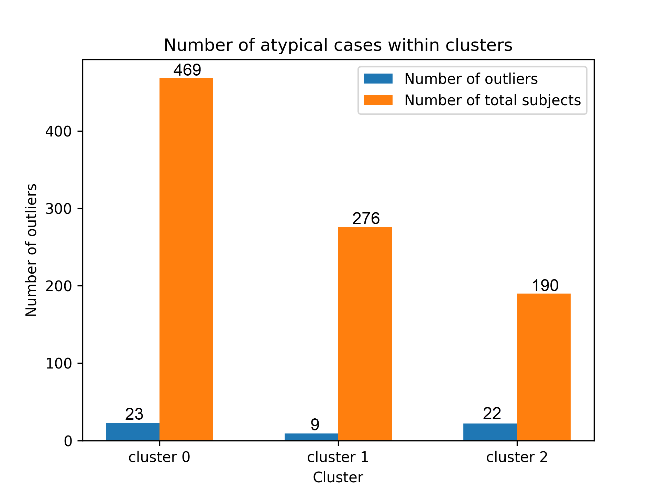

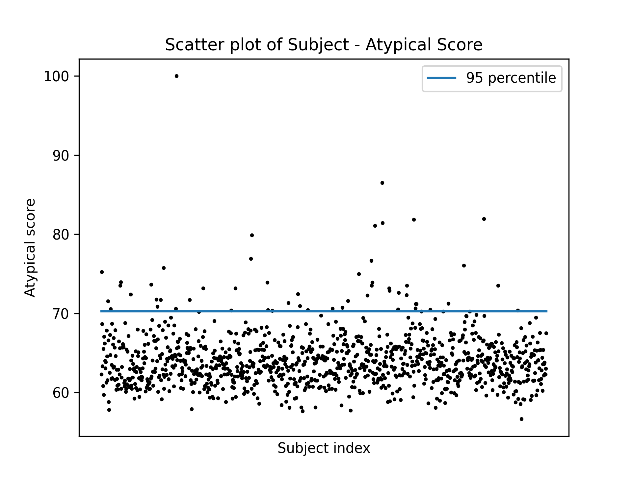


**A**

**B**

**C**


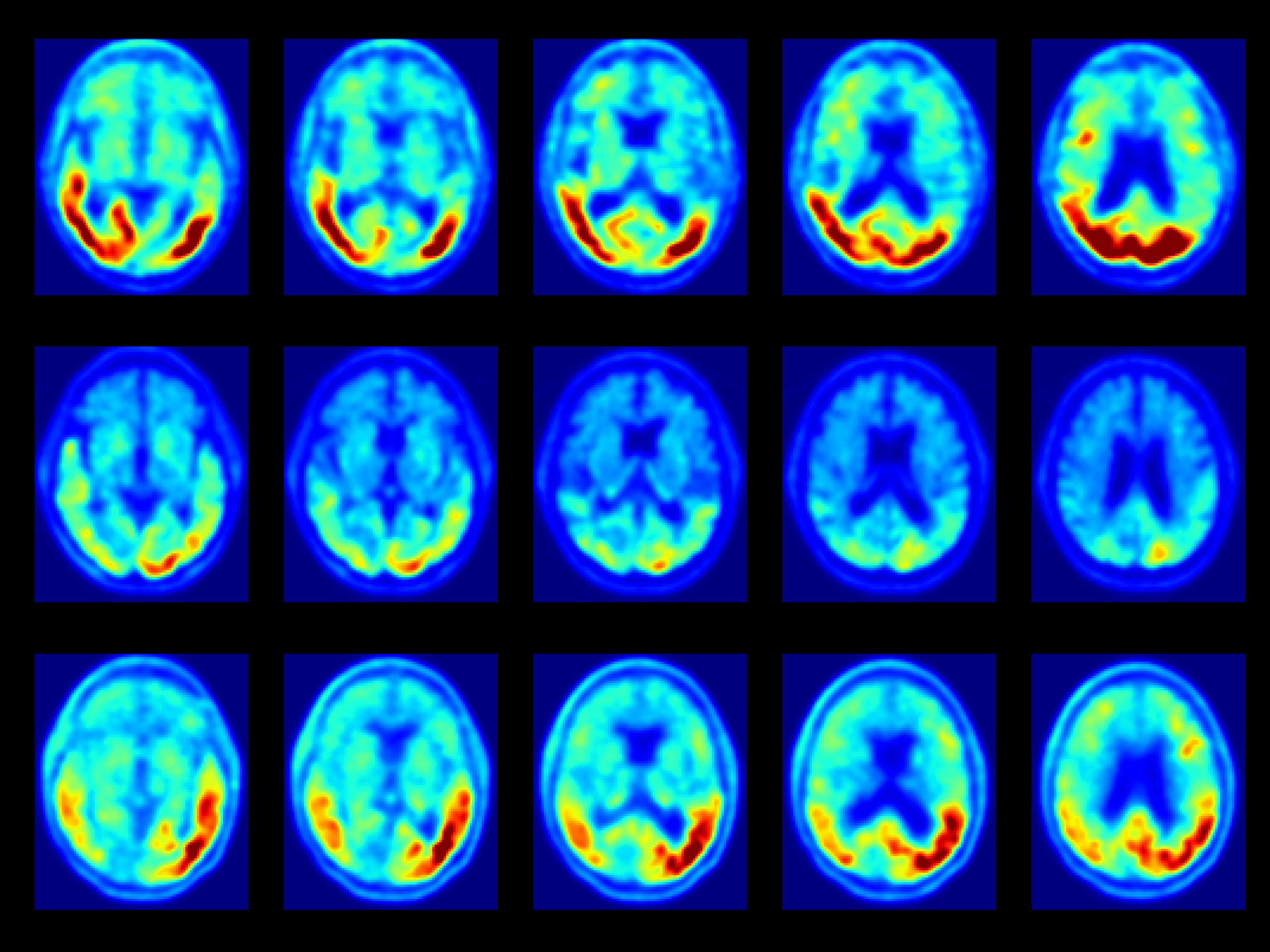

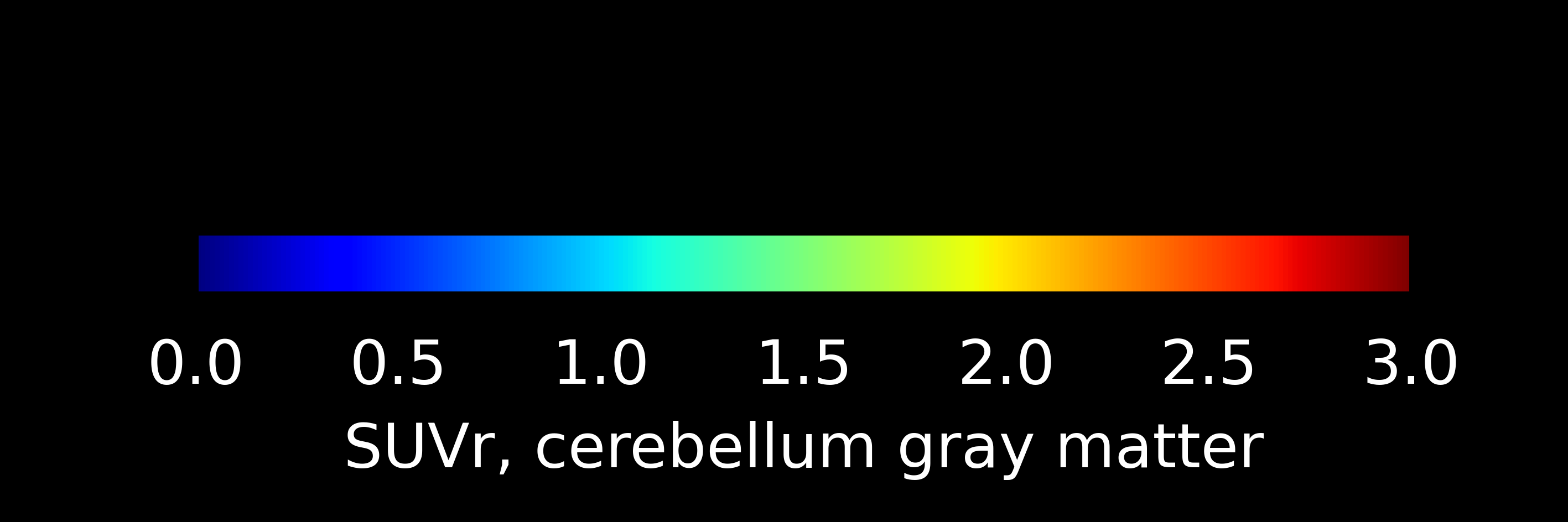


**Supplementary Figure 2. Atypical tau pattern detection.** **A.** Scatter plot of subjects – atypical score. The length between each latent feature and the respective edge in MST graph was calculated for each subject and scaled to 0-100, which is defined as an atypical score. The cut-off was set to 95 percentile (blue line) and outliers were identified, which are expected to exhibit an atypical tau pattern. **B**. Number of atypical subjects identified in each cluster. **C**. Three examples of the tau atypical patterns. Each row depicts the axial view of each subject.
